# Breeding and management of major resistance genes to stem canker/blackleg in *Brassica* crops

**DOI:** 10.1007/s00122-024-04641-w

**Published:** 2024-07-25

**Authors:** Paula Vasquez-Teuber, Thierry Rouxel, Annaliese S. Mason, Jessica L. Soyer

**Affiliations:** 1 Department of Plant Breeding, Justus Liebig University, Heinrich-Buff-Ring 26-32, 35392 Giessen, Germany; 2 Department of Plant Production, Faculty of Agronomy, University of Concepción, Av. Vicente Méndez 595, Chillán, Chile; 3 Plant Breeding Department, University of Bonn, Katzenburgweg 5, 53115 Bonn, Germany; 4 Université Paris-Saclay, INRAE, UR BIOGER, 91120 Palaiseau, France

## Abstract

Blackleg (also known as Phoma or stem canker) is a major, worldwide disease of *Brassica* crop species, notably *B. napus* (rapeseed, canola), caused by the ascomycete fungus *Leptosphaeria maculans*. The outbreak and severity of this disease depend on environmental conditions and management practices, as well as a complex interaction between the pathogen and its hosts. Genetic resistance is a major method to control the disease (and the only control method in some parts of the world, such as continental Europe), but efficient use of genetic resistance is faced with many difficulties: (i) the scarcity of germplasm/genetic resources available, (ii) the different history of use of resistance genes in different parts of the world and the different populations of the fungus the resistance genes are exposed to, (iii) the complexity of the interactions between the plant and the pathogen that expand beyond typical gene-for-gene interactions, (iv) the incredible evolutionary potential of the pathogen and the importance of knowing the molecular processes set up by the fungus to “breakdown’ resistances, so that we may design high-throughput diagnostic tools for population surveys, and (v) the different strategies and options to build up the best resistances and to manage them so that they are durable. In this paper, we aim to provide a comprehensive overview of these different points, stressing the differences between the different continents and the current prospects to generate new and durable resistances to blackleg disease.

## The history and importance of management of stem canker disease in *Brassica napus*

The *Brassica* genus contains numerous crop species, including turnip and Asian cabbages (*Brassica rapa*), broccoli, cauliflower and European cabbages (*Brassica oleracea*), rapeseed/oilseed rape/canola and swede (*Brassica napus*), and mustards (*Brassica nigra*—black mustard, *Brassica carinata*—Abyssinian mustard, *Brassica juncea*—Indian mustard) for which centuries of domestication have resulted in countless oilseed, leaf, root, and stem vegetable morphotypes. Related cultivated species include *Sinapis alba* (white mustard)*, Eruca sativa* (rocket), and *Raphanus sativus* (radish). These crops have been present in the human diet since ancient times. For instance, Greeks reported the use of mustard, cabbage, and kale in Europe before the common era, and in India *Brassica* crops first appeared around 2000 to 1500 before the common era (Bell 2012). The major crop species that are present in the genus *Brassica* comprise three diploid species: *B. nigra* (2*n* = 2*x* = 16 = BB), *B. rapa* (2*n* = 2*x* = 20 = AA), and *B. oleracea* (2*n* = 2*x* = 18 = CC), which hybridized to produce the allotetraploids *B. juncea* (2*n* = 4*x* = 36 = AABB), *B. carinata* (2*n* = 4*x* = 34 = BBCC) and *B. napus* (2*n* = 4*x* = 38 = AACC) (U 1935).

*Brassica napus* or rapeseed/oilseed rape/canola, the most economically significant crop in the *Brassica* genus, appeared a few thousand years ago in *Brassica* cultivation areas (reviewed by Mason and Snowdon 2016) and is recorded as a crop in Europe in the early middle ages (Gupta and Pratap 2007). Today, rapeseed is a crop used mainly for oil production, occupying the third place worldwide after palm oil and soybean, with world production still increasing (FAO 2023). The major producing regions are China, Canada, and Europe, with approximately 35 million hectares producing > 76 million tons in 2017, with yield increasing from 1.3 tons/ha in 1994 to 2.19 tons/ha in 2017. In Europe alone, production increased from 1.55 tons/ha in 1961 to 2.81 tons/ha in 2021 (FAO 2023).

*B. napus* as a crop is vulnerable to numerous insect pests, but relatively few diseases are of global importance: major, worldwide diseases include sclerotinia stem rot, clubroot, and stem canker (Zheng et al. 2020). Stem canker disease is caused by a complex of fungal species encompassing *Leptosphaeria maculans* and *Leptosphaeria biglobosa*. Worldwide, stem canker disease is estimated to cause annual losses of 10–15%, and in some cases up to 90% yield loss (Fitt et al. 2006; Sprague et al. 2006; Zheng et al. 2020). As is commonly observed for many crop diseases, especially those with aerial transmission, the outbreak of stem canker relies on a complex interaction between pathogen species, host, climate, and cultural factors, such as the use of fungicides, use of resistant plants, crop management, and rotations, physical distance between rapeseed crops, and accurate disease forecasting (West et al. 2001; Van de Wouw et al. 2021). Therefore, in the search for more sustainable rapeseed production, combinations of these strategies need to be applied to manage stem canker. Among the different strategies, the selection and breeding of resistant varieties offer a promising means to control stem canker disease incidence. The preferred control method is based on the use of cultivars possessing one or more major resistance genes coupled with quantitative resistance, which provides effective protection in a variety of environmental conditions (Delourme et al. 2006; Brun et al. 2010; Huang et al. 2018).

*B. napus* is unfortunately highly inbred, limiting the availability of resistance sources within this crop (Friedt et al. 2018). This is a result of its status as a recent allotetraploid species (originating from hybridization between only a few progenitor genotypes) as well as a result of intensive breeding for agronomic traits (Cowling 2007; Körber et al. 2012). No wild accessions of *B. napus* are known to exist which can be accessed to improve the genetic diversity present in this crop (Dixon 2006). However, the resynthesis of *B. napus* by crossing between varieties of the progenitor species *B. rapa* and *B. oleracea*, as well as hybridization with sister allopolyploids *B. juncea* and *B. carinata* which share the A and C genome with *B. napus*, respectively, is one option to transfer useful traits and diversity, as is the (more challenging) solution of hybridization with distant relatives (reviewed by FitzJohn et al. 2007; Mason et al. 2010; Katche et al. 2019; Quezada-Martinez et al. 2021).

The management of the disease necessitates consideration of the following points: the cropping cycle of the plant and its consequences on the fungal epidemiology, the fungal species responsible for the disease, the available resistance sources and their use in the past, and the structure and adaptability of pathogen populations. In all these aspects, major differences exist across different growing regions worldwide (Table 1) and the best resistance sources and their sustainable management may differ from one continent to the other, rendering solutions for one situation inapplicable for another.

**Table 1:** An overview of the characteristics of the disease due to *Leptosphaeria maculans* and the use of resistance genes in three major *Brassica napus* cropping areas where *Leptosphaeria maculans* is present

	Western Canada	Australia	France/Europe
*Plant genotypes and cropping practices*			
Plant genotypes grown	Spring types	Mostly spring types grown over winter	Winter types
Duration of the cropping season	3–4 months	5–7 months (11 months for “grain and graze” dual-purpose)	10–11 months
Specificities		Winter types grown as dual-purpose crops Mostly hybrids	Mostly hybrids No GM cultivars, no herbicide resistance
Environmental hazards	Long and cold winter season	Drought	Frost
*Stem canker/blackleg disease*			
First identification of *L. maculans*	1969 (Petrie 1969); 1975 (at Saskatchewan) (Dolatabadian et al. 2022)	1956 (Smith 1956)	1863 (Tulasne and Tulasne 1863)
First documented damages	1982 (at Saskatchewan) (Dolatabadian et al. 2022)	1972 (Roy and Reeves 1975)	1950s (Darpoux et al. 1957); 1964–1967 (Lacoste et al. 1969)
*Epidemiology*			
Primary inoculum	Ascospores and pycnidiospores (Zhang and Fernando 2018)	Ascospores	Ascospores
Importance of secondary inoculum	Common	Probably uncommon	Very low (West et al. 2001)
Duration of inoculum production	Just before and at flowering time (May–June)	Throughout the growing season (West et al. 2001)	Throughout the growing season (West et al. 2001)
Leaf spots	At flowering (June-July) (West et al. 2001)	Throughout the growing season (West et al. 2001)	Throughout the growing season (West et al. 2001)
Specificities of the disease	Seedling blight documented	Seedling blight documented Upper canopy infections (Amas et al. 2021)	No seedling blight
Survival of the fungus on residues	2–4 years	> 4 years	2–3 years (West et al. 2001)
Association with *L. biglobosa*	*L. biglobosa* ‘canadensis’; common but with variability depending on location (Dilmaghani et al. 2009)	*L. biglobosa* ‘canadensis’, ‘australensis’, ‘occiaustralensis’: rare (Vincenot et al. 2008)	*L. biglobosa* ‘brassicae’: very common; systematic association of the two species (Jacques et al. 2021)
*Control of the disease*			
First documented use of resistance genes	1995 (*Rlm3*) (Zhang et al. 2016)	1975 (*Rlm4*) (Rouxel et al. 2003b; Roy and Reeves 1975)	1970 (*Rlm2*) (Rouxel and Balesdent 2017)
Other resistance genes used in commercial varieties	*Rlm1* or *LepR3*, *Rlm4*, *RlmS-LepR2* (Zhang and Fernando 2018; Cornelsen et al. 2021)	*Rlm3*, *RlmS-LepR2*, *Rlm1* or *LepR3*, *LepR1*, *Rlm6*, *Rlm7* (Marcroft et al. 2012; Van de Wouw and Howlett 2020)	*Rlm1*, *Rlm3*, *Rlm4*, *Rlm7*, *Rlm9*, *RlmS-LepR2*, *LepR1* (Gautier et al. 2023; Balesdent and Jiquel, unpublished)
Breakdown of resistance gene	*Rlm3*: continuous between 1997 and 2010 (Zhang et al. 2016)	*LepR3* + *RlmS-LepR2*: 2003 (Sprague et al. 2006)	1972 (*Rlm2*); 1999 (*Rlm1*); *c.* 1999 (*Rlm4*) (Rouxel and Balesdent 2017); 2022 (*Rlm7*) (Balesdent et al. 2022)
Successive/concomitant use of *Rlm* genes	Successive/Concomitant	Concomitant	Successive
Maintenance of defeated *Rlm* genes in commercial varieties	Yes	Yes	Yes
Knowledge of *Rlm* gene content in varieties	Yes	Yes	Yes (recently only)
Communication toward farmers on *Rlm* gene content	Yes: resistance groups with R gene content (Canola Council of Canada 2021)	No: resistance groups with no information on resistance genes (Van de Wouw and Howlett 2020)	Yes: information on the presence of resistances showing efficiency (*Rlm3*, *Rlm7*, *RlmS-LepR2*) in each new variety (https://www.terresinovia.fr/colza)
Use of fungicides	Episodic	Systematic (Van de Wouw et al. 2021)	Uncommon

## Biology and epidemiology of *L. maculans* and the *L. maculans*-*L. biglobosa* species complex

Two *Leptosphaeria* species, *L. maculans*, and *L. biglobosa*, only discriminated in 2001 (Shoemaker and Brun 2001), are associated with stem canker disease (Rouxel and Balesdent 2005). One, *L. maculans*, is monomorphic, thought to be of recent origin (Dilmaghani et al. 2012), and reported from all *B. napus*-growing regions of the world, except China (Cai et al. 2018). The other, *L. biglobosa*, is divided into many subspecies and reported from all *B. napus*-growing regions with geographic specificities (Mendes-Pereira et al. 2003; Vincenot et al. 2008). In many parts of the world, the two species are found together in fields and individual plants at each stage of the disease (West et al. 2001; Jacques et al. 2021). *L. biglobosa* is the only causal agent of the disease in China, where it has been suggested to cause upper stem lesions and stem canker resulting in seed yield losses ranging from 10 to 37% (Cai et al. 2018). In all other parts of the world, however, *L. biglobosa* is considered a minor contributor to final yield losses compared to *L. maculans*. In addition, *L. biglobosa* has purely necrotrophic behavior (Lowe et al. 2014), and no difference in susceptibility/resistance has been documented so far between *Brassica* cultivars. Consequently, breeding for resistance has to date been targeted solely at *L. maculans*, with evident successes impacting disease outcomes and reducing yield losses, at least over short periods (Balesdent et al. 2022).

### History of the disease on different continents

Early mycologists described fungal species on the basis of morphological criteria that differ between the sexual and asexual forms of the species. This resulted in multiple distinct species names for a single species and the need to scrutinize historical records to retrace the history of these fungal species. For *L. maculans*, Tode in 1791 was the first to describe a fungal species associated with stem diseases of cabbages, named at the time *Sphaeria lingam* (cited in Henderson 1918). Subsequently, the authors independently described both the asexually reproducing stage of the fungus (the most popular name being *Phoma lingam*) and the sexual stage (*L. maculans*). In France, *L. maculans* and *P. lingam* were identified as two separate species as early as 1863 (Tulasne and Tulasne 1863). It was only during the twentieth century that experimental evidence that *L. maculans* and *P. lingam* are anamorphs of the same fungal species was obtained in Europe and other parts of the world: in the 1950s in Continental Europe (Müller and Tomasevic 1957), Australia and New Zealand (Smith 1956), and in the 1960s in the UK (Smith and Sutton 1964) and Canada (Petrie 1969), thus substantiating the presence of *L. maculans* in these parts of the world. The fungus was initially described on different crucifer species, and the first damaging epidemics were described in cabbage in the USA (Henderson 1918). However, it was only with the success of rapeseed as a major crop species, following years of breeding for improved agronomic traits coupled with subsequent narrowing of genetic diversity, that the first stem canker epidemics were recorded. As the first region to breed and grow rapeseed as a major crop (1938 in France for example), continental Europe was the first region of the world to be impacted by stem canker disease. The first epidemics were described in the 1950s in France (Darpoux et al. 1957) and caused average yield losses of around 40% between 1964 and 1967 (Lacoste et al. 1969; Table 1). In Australia, oilseed rape was first grown commercially in 1970 (in Western Australia), but in 1972, blackleg impacted crop yields so severely that less than 15 500 ha was sown between 1974 and 1991 (Roy and Reeves 1975; Sivasithamparam et al. 2005). In Western Canada before the 1970s, the disease, due to the sole presence of *L. biglobosa*, caused only very minor yield losses. *L. maculans* was first identified in Saskatchewan in 1975 (McGee and Petrie 1978), and later in Manitoba, Alberta, and British Columbia (Gugel and Petrie 1992). However, the first significant damages documented in Saskatchewan were in 1982, with yield losses up to 56% in some fields (Dolatabadian et al. 2022; Table 1).

### Pathogenic cycle and epidemiology of *L. maculans*

In Europe, the disease cycle starts with airborne ascospores produced on contaminated stubble (West et al. 2001; Rouxel and Balesdent 2005; Table 1). These ascospores land on cotyledons and leaves, where they germinate rapidly and the resulting hyphae penetrate the leaf through stomata or wounds (West et al. 2001). If the plant is susceptible, the hyphae first grow slowly in the apoplast and then massively colonize the intercellular spaces around the inoculation point to eventually cause grayish-green leaf spots on which the asexual organs, the pycnidia, differentiate (West et al. 2001). The pycnidiospores produced are of very limited importance in the disease cycle (at least in Europe) due to continuous production of ascospores throughout the plant vegetative cycle, low dispersal ability (rain-splash dispersal), and low infectivity of the pycnidiospores (Rouxel and Balesdent 2005). Concomitantly with leaf spot development, the mycelium heads to the petiole and travels down the stems asymptomatically (Hammond et al. 1985; Huang et al. 2014). In the intercellular spaces of the stem tissues, the fungus maintains low transcriptional activity for a few months, during winter, and “revives” in spring with the initiation of waves of expression of genes associated with stem biotrophy (Gay et al. 2021). Eventually, it turns necrotrophic again and expresses a stem base canker (“crown canker”). In severe cases of stem canker, complete lodging and death of the plants occur (West et al. 2001). The fungus then lives as a saprobe on stem residues where it differentiates pseudothecia, producing the ascospores (West et al. 2001; Rouxel and Balesdent 2005).

In the three main growing regions of the world (excluding Asia, where no *L. maculans* has been identified to date), the vegetative cycle of *B. napus* is different, explaining important differences in the epidemiology/disease cycle of *L. maculans* between Europe, Western Canada, and Australia (West et al. 2001; Table 1). Western Canada exclusively grows spring-type varieties (canola) with a very short vegetative cycle (3–4 months between May and August). These varieties have very few resistance genes to *L. maculans* and a low *R* gene diversity (Rouxel et al. 2003a; Cowling et al. 2007). At the other extreme, Europe mainly grows winter-type rapeseed, necessitating vernalization to bloom, with a very long (10–11 months) vegetative cycle, from early August to July (Table 1). These winter cultivars harbor a limited variety of resistance genes (“R” genes), but, in contrast with spring varieties, genotypes devoid of resistance genes to *L. maculans* are rare (Rouxel et al. 2003b). In Australia, the situation is more complex, with mostly spring types grown for an extended time (5–7 months from March to September), but also winter types for dual purpose used firstly for grazing livestock and then for seed production. These latter types have a long vegetative cycle (11 months; Van de Wouw et al. 2021).

The *L. maculans* disease cycle is complex and strongly depends on the vegetative cycle of the host plant (see Rouxel and Balesdent 2005; Gay et al. 2021; Dolatabadian et al. 2022; for a diagram of the *L. maculans* lifecycle on *B. napus*). In particular, the pathogenic cycle has the specificity to necessitate an early entry point in leaves, followed by systemic colonization of the plant stem, eventually resulting in stem canker a few weeks before harvest. Thus, while leaf spots usually appear continuously throughout the growing season (West et al. 2001) in Europe or Australia, they only are present at flowering time in Western Canada, leaving a very short time for the fungus to proceed to the stem and develop the cankers which are a prerequisite for differentiation of pseudothecia. In this respect, there are still debates in the literature on the relative importance of pycnidiospores and ascospores to initiate and propagate the disease in Western Canada, and leaf spots may be unnoticed before stem canker develops (Zhang and Fernando 2018). This would be in accordance with similar findings on short vegetative cycle cabbages, on which only pycnidia differentiate and clonality of the *L. maculans* population is the rule (Dilmaghani et al. 2012). However, no signs of clonality have been identified in populations isolated from Western Canada on canola (Dilmaghani et al. 2009), and the authors claim that pycnidiospores are important contributors to the disease cycle in Australia (Li et al. 2007) or even the main inoculum source in Western Canada (Zhang and Fernando 2018). However, further research is required to support this claim with epidemiological studies.

In Europe, the stem canker is essentially a monocyclic disease (the secondary inoculum, pycnidiospores, only have a very limited contribution to the epidemiology) (West et al. 2001). Pseudothecia differentiate on leftover residues and remain present throughout the growing season, even for many years (up to four in Australia), as long as the woody residues remain (West et al. 2001). This has possible consequences in terms of quality and latency of inoculum production. In all cases, ascospores are the primary inoculum and are released under strict climate conditions requiring continuous wetting of the residues by rain for maturation (Toscano-Underwood et al. 2003), and, at least in Europe, a concomitant drop in temperature (Peres et al. 1996). Pseudothecial maturation is delayed by dry hot summers in Western Australia and by sub-zero winter temperatures in Western Canada, allowing synchronicity between maturation and sowing even though the delay between harvest and next sowing is very long in Australia and even more in Western Canada (9–10 months after harvest) (West et al. 2001). In Europe, mature pseudothecia can be observed at sites of severe cankers on stem bases shortly after harvest (West et al. 1999). The period of ascospore release varies from region to region but usually coincides with the presence of young, susceptible plants. In Australia and Canada, intense and early ascospore release can cause seedling blight. Ascospores are then produced and released throughout the growing season (Table 1), and there is a clear correlation between the intensity of ascospore discharge and the number of leaf spots (Peres et al. 1996). Continuous ascospore discharge, and changing practices such as early sowing, may also result in yield-limiting spotting on siliques (i.e., upper canopy infection), detected in Australia since 2010. While this symptom has been described occasionally, e.g., on cabbage (Bonman et al. 1981), it is becoming a concern in Australia with reductions in grain yield up to 30% (Van de Wouw et al. 2021). While the link between ascospore discharge and leaf spotting is evident (whenever no efficient major gene operating at the leaf level is used), the continuity between earliness/intensity of leaf spotting and severity of stem canker is often questioned, since many factors influence this relationship (duration of cropping, environmental conditions, genotypes used, involvement of other diseases or insect damages, etc.). For example, cultivars susceptible at the leaf spotting stage may be devoid of stem symptoms (Thurling and Venn 1977). The reverse may also be found, i.e., the absence of leaf spotting but severe cankers at the end of the growing season (mainly observed in Canada). However, absence of leaf spotting due to the influence of major effect resistance genes usually results in a low level of stem cankers at the end of the growing season (Borhan et al. 2022).

One other important difference in cropping practices to control the disease lies in the use of fungicides, with a constantly increasing use of fungicides at sowing and during the vegetative growth stage in Australia (Van de Wouw et al. 2021), episodic use of fungicides in Western Canada, where the foliar application of fungicides is thought to provide only a limited yield benefit, except when applied to highly susceptible genotypes (Dolatabadian et al. 2022), and no or limited use of fungicides in Europe (Table 1). Thus, the reliance on genetic resistance to control the disease ranges from very high in continental Europe to moderate in Australia, with a somewhat intermediate situation in Canada.

## Resistance to *L. maculans* in *Brassica* species

### Bases of plant resistance to infection

Plant pathogens and their hosts have undergone a long co-evolution, such that pathogens become adapted to either a few hosts (specialized pathogens) or numerous hosts (generalist pathogens) (Corwin and Kliebenstein 2017). Most interactions between a host and a potential pathogen fail, either because the pathogen is not adapted to infect the host plant or because the plant species has an array of mechanisms to defend itself. The first barrier to be overcome by a potential pathogen is physical/chemical: for example, leaf topography, or the intrinsic presence of chemicals or compounds that pathogens cannot detoxify. Once these barriers are overcome, the pathogen encounters the first immune resistance layer, involving the recognition of pathogen-conserved molecular motifs (such as fungal chitin), and is called Pathogen/Microbe-Associated Molecular Pattern (P/MAMP) initiated by plant receptors called Pattern Recognition Receptors (PRR). This recognition triggers basal defenses, known as PAMP Triggered Immunity (PTI), including mechanisms such as stomatal closure to avoid pathogen colonization, and the production of general plant defense compounds. In the course of co-evolution, some pathogen species became able to overcome this recognition via the production of effectors. Effectors are either secondary metabolites, small RNAs, or the well-studied class of small secreted proteins which suppress the host immune system or manipulate its cell physiology to favor penetration and colonization (Lo Presti et al. 2015). The host, in turn, may set up a defense system involving direct or indirect recognition of effectors by “resistance” proteins, encoded by resistance (*R*) genes and activating inducible defenses. Proteinaceous effectors specifically recognized by host resistance proteins are then called Avirulence (Avr) proteins. This is referred to as qualitative resistance (depending on a monogenic determinism), as opposed to quantitative resistance (involving a polygenic control). The recognition between avirulence and resistance proteins activates the second layer of immunity, called Effector Triggered Immunity (ETI), which results in local cell death: this is generally in the form of a hypersensitive reaction (HR), which prevents the pathogen from spreading further. This interaction is referred to as the gene-for-gene model, in which for a given resistance gene on the host side there is a corresponding avirulence gene on the pathogen side (Flor 1955). Typically, in the literature, most plant *R* genes are said to encode intracellular nucleotide binding site leucine-rich repeat (NLR, also known as NBS-LRR) proteins, while PAMP-triggered immunity is generally associated with the recognition of M/PAMPs by membrane-associated Receptor-Like Proteins (RLP) or Receptor-Like Kinases (RLK) (Tang et al. 2017; Ngou et al. 2022). This somewhat simple delineation was mainly because most research in molecular plant–pathogen interaction has focused on models involving intracellular pathogens or pathogens that inject actively their effectors in the plant cell. This has changed when addressing apoplastic pathogens such as *L. maculans* or *Zymoseptoria tritici* for which all currently known *R* genes are cell surface receptors, and some authors proposed to abandon the PTI/ETI denomination and change it to “surface-mediated immunity” (SRMI) vs. “intracellular receptor-mediated immunity” (IRMI) (Ding et al. 2020). Pathogens can in turn overcome effector-triggered immunity by deletion or mutation of the *Avr* gene. The circumvention of this monogenic resistance is facilitated by biological factors (e.g., population size, reproduction mode) or genomic characteristics (e.g., location of genes encoding avirulence proteins in repeat-rich genomic regions, dispensable chromosomes, subtelomeric regions, or large transposable element (TE)-rich regions) (Sánchez-Vallet et al. 2018). In contrast, quantitative resistance is more difficult to circumvent by the pathogen since it relies on several loci (Pilet-Nayel et al. 2017; Niks et al. 2015).

### Overview of the resistance to *L. maculans* in *Brassica* species

Historically, at least in Europe where breeding for resistance to *L. maculans* began, plant resistance was evaluated in the field at the end of the long rapeseed cropping season. The plants were thus submitted to a combined load of biotic and abiotic stresses, including mixed avirulent and virulent pathogen populations. Under these conditions, it was virtually impossible to dissect genetic mechanisms underlying resistances, and breeders generally assumed the crop expressed “adult-stage”, multigene, quantitative resistance (e.g., Cargeeg and Thurling 1979). However, some authors postulated very early on that there may be differential behavior of plant genotypes faced with different populations of the fungus, despite still supporting a system of polygenic control/interaction on both the plant and the pathogen side (Thurling and Venn 1977). It was only from the 1990s onwards that studies began to be performed on the fungal side to dissect host specificity and that rapid miniaturized inoculation tests on plantlets became available, enabling the different components of *Brassica* resistance to *L. maculans* to be dissected. Subsequently, it was demonstrated that qualitative “gene-for-gene” resistance operates in this species as well as quantitative resistance. As many recent reviews have nicely summarized the findings on quantitative resistance of *Brassica* spp. toward *L. maculans* (Amas et al. 2021; Borhan et al. 2022), this paper focuses on critical questions regarding identification, uses, and prospects for durability of qualitative resistances in the *L. maculans*/*Brassica* pathosystem.

## Methods for identifying qualitative (major effect/gene-for-gene) resistances in the *L. maculans-B. napus* pathosystem

### The cotyledon inoculation test

To bypass the tedious process of field-based assessment of resistance, researchers began to design miniaturized tests to allow screening of a large number of *Brassica* and *L. maculans* genotypes under controlled conditions, in conjunction with the development of reliable scoring methods to assess symptoms. Trials encompassed the use of ascospore suspensions obtained from mixed field residues inoculated on cotyledons without wounding and assessment of cotyledon and stem lesions (Thurling and Venn 1977), single ascospore inoculation of cotyledons (Cargeeg and Thurling 1979), blended mycelium inoculation of cotyledons without wounding (Cargeeg and Thurling 1979), inoculation of the first leaf petiole with paper disks soaked in pycnidiospore suspension after wounding (Newman 1984), inoculation of the lamina of the first true leaf with mixed pycnidiospore suspension after wounding (Mithen et al. 1987), and cotyledon inoculation test with pycnidiospore suspension from a single isolate after wounding (Mengistu et al. 1991; Badawy et al. 1991; Travadon et al. 2009). McNabb et al. (1993) compared the accuracy of four different inoculation tests: cotyledon inoculation with cotyledon rating, leaf inoculation with leaf and stem rating, stem inoculation with stem rating, and inoculation using infested oat kernels with stem rating. They concluded that the highest reliability (in terms of reproducibility and positive correlation with field behavior) was attained with the cotyledon test. There have been endless debates on the best inoculation tests to identify relevant *R* genes, starting from the pioneering work of Mengistu et al. (1991) using a simple inoculation test and a defined rating scale. While a few authors were initially dubious (Newman and Bailey 1987), numerous others subsequently agreed on the high reliability of miniaturized cotyledon tests, which were then rapidly adapted for breeding programs (McNabb et al. 1993).

One of the issues with using cotyledon inoculation tests to identify new sources of resistance is that field isolates of *L. maculans* generally harbor numerous *Avr* genes, rendering it complex to identify if a novel resistance is present in a given genotype. After Mengistu et al. (1991, 1993) identified three *B. napus* genotypes with different pathogen responses and set up protocols for in vitro crosses of the fungus, crosses between different fungal isolates under laboratory conditions (Gall et al. 1994) demonstrated that the avirulence phenotypes identified in two *B. napus* genotypes (cvs. ‘Quinta’ and ‘Glacier’) were under monogenic control in the fungus. This led to the characterization and subsequent cloning of two effector genes: *AvrLm1* (avirulence against ‘Quinta’; Ansan-Melayah et al. 1995) and *AvrLm2* (avirulence against ‘Glacier’; Ansan-Melayah et al. 1998). These genetic analyses strongly suggested that major resistance genes (*Rlm*) corresponding to avirulence genes existed in rapeseed, which was then validated via genetic analyses on the plant side (Ansan-Melayah et al. 1998). Combining expertise from plant geneticists, plant pathologists, and fungal geneticists, more than 10 other avirulence genes were characterized in *L. maculans* and the corresponding resistance genes identified in oilseed rape (*Rlm1*, *Rlm2*, *Rlm3*, *Rlm4*, *Rlm7*, *Rlm9*), as well as in other *Brassica* species such as Indian mustard, *B. juncea* (*Rlm5*, *Rlm6*), black mustard, *B. nigra* (*Rlm10*) and turnip rape, *B. rapa* (*Rlm1*, *Rlm3*, *Rlm7*, *Rlm8*, *Rlm11*, *RlmS-LepR*2, *LepR1*, *LepR4*) (Rouxel and Balesdent 2017; Ghanbarnia et al. 2012; Neik et al. 2022). Due to the complexity of *AvrLm* genes harbored by field isolates, crosses of the fungus were the first option to generate isolates harboring the smallest possible number of *AvrLm* genes and, following a series of backcrosses, near-isogenic isolates differing by only a single *AvrLm* gene (Balesdent et al. 2002; Huang et al. 2010; Rouxel et al. 2003b). With the advent of molecular tools, genetic manipulation methods (complementation, RNA silencing of gene expression, and CRISPR-Cas9 gene inactivation) are now routinely used as tools to generate isogenic isolates to identify corresponding *Rlm/LepR* genes in *Brassica* genotypes, for screening genetic resources, or for use in plant breeding (Balesdent et al. 2002; Rouxel et al. 2003b; Ghanbarnia et al. 2012; Van de Wouw et al. 2014; Larkan et al. 2015).

### Problems with resistance gene identification: nomenclature and availability of genetic resources

Over the years, considerable confusion in resistance gene naming has become evident, with independent naming of genes by independent research groups working on the *L. maculans*/*Brassica* spp. pathosystem. This phenomenon is by no means restricted to this pathosystem and is also found for instance in the rice–*Magnaporthe oryzae* model (Ballini et al. 2008). This terminology confusion can be attributed to the difficulty in developing and sharing common sets of differential pathogen strains, as well as plant genotypes with an identified and fixed set of resistance genes. In the case of differential isolates in particular, this has been slowed down, or even made fully impossible, by import restrictions established in some parts of the world, which have banned the import of biological material that could be used to demonstrate the identity of a resistance gene in geographically or genetically distant plant material (e.g., in Australia). Considerable difficulty is also involved in tracing and validating the identity of plant genotypes available to the community. Additionally, the initial identification of resistance loci was performed before the *AvrLm*-*Rlm* gene-for-gene interaction was understood and considered as the rule in this system, and the isolates used for the identification of resistance loci were either field populations or ill-defined isolates in terms of avirulence gene content (Delourme et al. 2006). Some groups even complexified the miniaturized tests by inoculating with mixes of isolates that may be representative of the diversity of their local populations (Gaebelein et al. 2019).

Nowadays, most of the groups studying the *L. maculans*–*Brassica* interaction use a common *AvrLmX*-*RlmX/LepRX* terminology. The generation of progeny isolates harboring only a limited and known content of characterized effector/avirulence genes has helped to characterize the resistance gene complement of some of the genotypes that were previously employed, and the transposition of markers from one genetic map to another has allowed, with time, previously described resistance genes to be concatenated and assigned (e.g., *LEM1*, *LmFr1*, *LmR1*, *ClmR1,* assigned to chromosome A07 of *B. napus*, were firstly assumed to all correspond to *Rlm4* based on map position and phenotypic responses; Rouxel et al. 2003b; Rimmer 2006; and more recently were shown to all actually correspond to *Rlm3*; Larkan et al. 2016a). The availability of reference genomes and the possibility to physically localize *Rlm* and *AvrLm* genes cleared up a lot of initially confusing results from early linkage mapping studies, where the same gene was often repeatedly detected or where different genes were occasionally conflated (Borhan et al. 2022). Despite these improvements, confusion persists when identifying new resistance or avirulence genes, exemplified by the recent cloning of two avirulence genes in two different research groups, which turned out to be the same gene (i.e., *AvrLmS-Lep2* or *AvrLmJ1*/*AvrLm5-9*; Van de Wouw et al. 2014; Plissonneau et al. 2017; Neik et al. 2022).

Additional confusion arises when genotypes with the same name harbor different complements of *Rlm* genes, which is sometimes the case when the initial cultivar was not a fixed line, and as such may contain individual plants with different *Rlm* gene complements. Further independent self-pollination events may thus independently fix different *Rlm* genes in different locations (for instance, cv. Tina, with *Rlm3*, *Rlm7,* or no *Rlm* genes present, was used to derive differential lines with *Rlm3* or *Rlm7* (Balesdent et al. 2002), while cv. Quinta could harbor *Rlm1*, *Rlm3* and/or *Rlm4*). This issue of genetic divergence between accessions with the same name may also occur due to the high frequency of unintended outcrossing present in *Brassica* species, an issue long recognized by breeders and researchers. This is an ongoing problem, and although solutions such as a registered accession database have been suggested and may be implemented in time (e.g., Yang et al. 2023), to date most research groups simply reconfirm resistance gene presence via cotyledon testing with specific isolates after receipt of accessions rather than trusting to published information. However, this additionally emphasizes the importance of coordination in efforts by the international community to identify resistance or avirulence genes.

### Progress in resistance gene identification

If considering only genes for which a map position has been defined, or for which an avirulence gene has been cloned, at least 18 resistance genes that operate at the cotyledon/leaf stage are known for the *L. maculans*–*Brassica* pathosystem, and one additional gene, *RlmSTEE98*, that operates in the stem (Table 2). Five of these, corresponding to only two distinct loci, have been cloned (Larkan et al. 2013, 2015, 2020). Most resistance genes described so far in the A genome of *B. napus* are also present in the A genome of *B. rapa*, such as *Rlm1*, *Rlm2* and *Rlm7* (Leflon et al. 2007). One gene, *Rlm13*, was mapped to chromosome C03 in *B. napus* but is likely a translocation from the homologous region of A03 (Raman et al. 2021). Few resistance genes have been identified in *B. oleracea* (C genome) with only two described or postulated to date, *Rlm13* and *Rlm14* (Raman et al. 2021; Degrave et al. 2021). In contrast, a higher number of resistance genes have been identified in diverse *B. rapa* germplasm (*Rlm1*, *Rlm2*, *Rlm7*, *LepR1-4*, *Rlm11*). However, some of these may be identical to those which were previously identified (*RlmS* and *LepR2*, renamed *RlmS-LepR2*; Neik et al. 2022) or may be functional homologs recognizing the same avirulence gene (e.g., *Rlm1* and *LepR3*, which both recognize *AvrLm1*; Larkan et al. 2013; Rouxel and Balesdent 2013). Fewer investigations have been made in other *Brassica* species of less agronomic interest and genetic similarity to *B. napus*, although *Rlm10* has been identified in *B. nigra* (Chèvre et al. 1996; Chèvre et al. 1997a, b), and *Rlm5* and *Rlm6* in *B. juncea* (Balesdent et al. 2002; Struss et al. 1991).

**Table 2:** Resistance genes identified in the *Leptosphaeria maculans* – *Brassica* spp. pathosystem

Gene	Species^a^	Genotype^b^	Chromosome^c^	Reference
*Rlm1*	*B. napus*	Quinta	A7	Ansan-Melayah et al. 1998
Rlm2*	*B. napus*	Glacier	A10	Ansan-Melayah et al. 1998; Larkan et al. 2015
*Rlm3*	*B. napus*	Glacier, Maxol	A7	Delourme et al. 2004
Rlm4*	*B. napus*	Jet Neuf	A7	Balesdent et al. 2002; Haddadi et al. 2022
*Rlm5*	*B. juncea*	Aurea, Picra	–	Balesdent et al. 2002
*Rlm6*	*B. juncea*	Aurea, 150-2-1 and 151-2-1	B8	Chèvre et al. 1997a
Rlm7*	*B. napus*	Caiman	A7	Balesdent et al. 2002; Haddadi et al. 2022
*Rlm8*	*B. rapa*	156-2-1	–	Balesdent et al. 2002
Rlm9*	*B. napus*	Darmor-*bzh*	A7	Delourme et al. 2004; Larkan et al. 2020; Haddadi et al. 2022
*Rlm10*	*B. nigra*	Junius	B4	Chevre et al. 1996
*Rlm11*	*B. rapa*	plant (2323) from line 02-159-4-1	–	Balesdent et al. 2013
*Rlm12*	*B. napus*	Skipton/Ag-Spectrum	A1	Raman et al. 2016
*Rlm13*	*B. napus*	ATR-Cobbler	C03	Raman et al. 2021
*Rlm14*	*B. oleracea*	Monaco	–	Degrave et al. 2021
*RlmS-LepR2*	*B. rapa*	Surpass 400	A10	Van De Wouw et al. 2009; Neik et al. 2022
*LepR1*	*B. rapa*	DHP95	A2	Yu et al. 2005
LepR3*	*B. rapa*	Surpass 400	A10	Li and Cowling 2003; Yu et al. 2008; Larkan et al. 2013; Larkan et al. 2014; Larkan and Bohran 2015
*RlmSTEE98*	*B. napus*	Yudal	A9	Jiquel et al. 2021

^*^indicates cloned *Rlm* genes; *Rlm4*, *Rlm7,* and *Rlm9* are allelic versions of the same gene; *Rlm2* and *LepR3* are allelic versions of the same gene

^a^Species in which the gene has been identified

^b^Genotype in which the resistance gene has been first identified

^c^Position of the resistance gene

Several resistances have also been putatively identified but not yet confirmed or mapped as single major genes. It has been suggested that *B. nigra* contains at least two resistance loci on different chromosomes (Rimmer and Van Den Berg 1992; Struss et al. 1996). In cross-progeny between *B. carinata* and *B. napus*, the presence of the middle to bottom of chromosome B3 and the top of B8 were independently associated with resistance (Fredua-Agyeman et al. 2014). Resistance has also been found in *B. oleracea* var. *capitata* Korean germplasm genotypes (Robin et al. 2017) and putatively in the C genome of the allotetraploid species *B. carinata* (Rahman et al. 2007). In addition, 12 putative *R*-genes were found in *B. oleracea* within a syntenic region of *LepR4* (Ferdous et al. 2020). While *B. napus* germplasm and, to a lesser extent, *B. oleracea* and *B. rapa* collections of genotypes have been comprehensively investigated to identify sources of resistance, this is much less true of the three mustard species *B. nigra*, *B. juncea,* and *B. carinata*, and it would be logical that this germplasm could also comprise a useful source of diverse new resistance genes, as was the case for *B. rapa*.

### Variability of phenotypic expression in miniaturized inoculation tests

Problems in resistance gene identification may arise from variations in the expression of disease symptoms. These may depend on the genetic background of the *B. napus* accession considered, differences in environmental conditions between locations in which the test is performed, stability of the environmental conditions over time, rating system, and interpretation of the scores. As mentioned previously, although the *Rlm*-*AvrLm* interaction is under monogenic control, macroscopic expression of the interaction phenotype can still vary. Under controlled conditions, resistance can sometimes result in a localized, complete, and rapid hypersensitive response (i.e., a small-sized black response at the site of inoculation): this is typical of the *AvrLm4*-*Rlm4* interaction in cultivars ‘Jet Neuf’ or ‘Pixel’, or the *AvrLm7*-*Rlm7* interaction in cultivars ‘Caiman’ or ‘DK Excellium’ (Blondeau et al. 2015). Alternatively, the phenotypic expression of resistance can sometimes be diffuse, with more extended necrosis or longer establishment period: this is typical of the *AvrLm1*-*Rlm1*, *AvrLm3*-*Rlm3,* and *AvrLmS*-*RlmS* interactions (Ansan-Melayah et al. 1995; Van De Wouw et al. 2009). The phenotypic responses can also differ depending on the host genotype. This phenomenon has been regularly reported in the *L. maculans*/*B. napus* pathosystem as well as in other pathosystems (Röhrig and Dussart 2022) and can involve factors such as putative heterozygosity vs. homozygosity, allelic variation, and the host genetic background in which the *R* gene is inserted. The first study to formally address the influence of the host genotype on major gene responses was undertaken in 2019, by Haddadi et al. They introgressed two susceptible genotypes, ‘Topas’ and ‘Westar’, with the same resistance gene (either *Rlm2*, *Rlm3*, *LepR1,* or *LepR2*), and assessed their phenotypic and transcriptomic behavior when facing an isolate of *L. maculans* having the cognate *AvrLm* genes. Inoculation of ‘Topas’ and ‘Westar’ resulted macroscopically in similar large lesions, while inoculation of ‘Topas’ or ‘Westar’ in which an *R* gene had been introgressed resulted in different macroscopic expression of resistance. The phenotypic assessment of resistance was mirrored by RNA-seq analysis at the same time points, under infection. This provided an overview of key genes and pathways mobilized in the resistance response, such as genes related to pathways involved in plant defense (salicylic acid, jasmonic acid, abscisic acid, ethylene, and glucosinolate pathways) which are expressed earlier in introgressed resistance lines compared to susceptible lines. Overall, the authors showed that although similar pathways were upregulated in all introgressed lines post-infection, ‘Westar’-introgressed lines showed delayed expression of the genes involved in plant defense compared to ‘Topas’-introgressed lines. This is the only comprehensive analysis to date of the influence of the host genetic background on phenotypic expression of resistance, and many variable resistance phenotypes remain elusive. This includes the expression of *Rlm7* in the first registered cultivar harboring this resistance, ‘Roxet’, which is still the only case known in which resistance is not expressed as a typical hypersensitive response. Whether such phenomena may be due to a co-dominant HR response for *Rlm7* when mapped in ‘Roxet’ (Larkan et al. 2016a), heterozygosity, to less efficient variants of the resistance gene, or an unrelated gene recognizing also *AvrLm4-7* remains to be elucidated.

A second source of variation is likely due to the environmental conditions in which the pathogenicity assay is performed. Temperature and wetness influence the phenotypic expression of both major gene and quantitative resistances (Huang et al. 2006, 2009; Larkan et al. 2016b; Neik et al. 2022). An increase in temperature is associated with a shortening of period before the first symptoms appear on the leaf, and wetness also influenced the number of lesions observed irrespective of genotype (Huang et al. 2009). Recently, the influence of temperature on the expression of the hypersensitive response was also investigated by two different groups (Yang et al. 2021; Noel et al. 2022). Yang et al. (2021) tested the expression of the *B. napus*/*L. maculans* interaction under different temperature conditions and showed that lesion size was larger at elevated temperatures and that this was consistently underpinned by differences in the expression of genes associated with plant defense and response to temperature. Noel et al. (2022) also monitored the expression of quantitative resistance in field experiments as well as under controlled conditions, showing that although some cultivars exhibit robust resistance under high temperatures, resistance efficacy may decrease during long heat waves. A better understanding of plant resistance under various environmental conditions is an important field of research to consider in the context of global warming (Röhrig and Dussart 2022).

Of major concern is that variation in the resistance response (or its interpretation) may be observed for a given interaction when tests are performed at two different locations with the same control isolate and the same batch of seeds, all freshly shared between two groups, as shown in the study of Neik et al. (2022). One of the hypotheses to explain the variable expression of symptoms and contrasting interpretations of the interaction was a strong influence of environmental conditions on the phenotypic outcome, a hypothesis that remains to be validated. In this specific example, it is interesting to note that despite the divergent interpretation of the phenotypes, the use of crosses involving isolates with contrasting phenotypes on Topas-*LepR2* nevertheless allowed for independent cloning of the corresponding avirulence gene *AvrLmS-Lep2*. Use of cultivars like ‘Topas’, a Canadian line now considered as a universally susceptible genotype (and the source of isogenic lines widely shared in the community; Larkan et al. 2016a), but with a general level of resistance higher than the previously used ‘Westar’ (McNabb et al. 1993), may also result in confusing scores between susceptible and resistant interactions when using European isolates. Whether this is due to an adaptation of Canadian populations to this widely grown cultivar in Canada and a contrastingly poor adaptation of European isolates remains to be investigated. The complexity of the genetic background and environmental interactions should thus be considered carefully in experiments designed to identify novel sources of resistance.

## Molecular insights into *AvrLm* and *Rlm* genes

### Genomics methods for candidate resistance gene prediction

Resistance genes can be categorized using their conserved domains and structural features into the three major families of Resistance Gene Analogs (RGA; for review see Sekhwal et al. 2015): nucleotide binding site leucine-rich repeats (NBS-LRR), receptor-like kinases (RLK), and receptor-like proteins (RLP). The advent of the genomics era afforded great progress in the elucidation of the genetics underlying the *L. maculans*-*Brassica* pathosystem (Cantila et al. 2021) together with in silico prediction of thousands of RGA in plant genomes. Due to an increase in available plant genomes and pangenomes, attempts have been made to take a “bottom-up” in silico approach to *R* gene discovery, working from existing knowledge of expected functional domains in *R* genes (Kruijt et al. 2005) or based on homology to genes previously established to be involved in resistance responses. Using these approaches in *Brassica* spp., *Rlm* genes have been mined from the pangenome of *B. oleracea* and in several other Brassicaceae species, including *Arabidopsis thaliana* (e.g., Bayer et al. 2019; Dolatabadian et al. 2020; Cantila et al. 2022; Amas et al. 2023). Resistance gene analog prediction in *B. oleracea* based on pangenome analyses has highlighted that resistance gene content is highly divergent between accessions and that receptor-like kinases may be the most important class of *Brassica* resistance genes. Transposable elements (TE) and presence/absence gene variation have also been identified as important drivers in generating novel disease resistance and diversification of putative resistance genes (Bayer et al. 2019), showing that a pangenome approach is essential to fully describe and assess the diversity of resistance genes within a species. In *B. napus*, available genomic resources have expanded tremendously in the last decade, from the initial publication of the genome sequence of ‘Darmor-*bzh’* (Chalhoub et al. 2014) to the first pangenome based on analysis of eight accessions (Song et al. 2020) and the availability of datasets representing genotyping data from c.a. 1,700 accessions (Song et al. 2021) (BnPIR database: http://cbi.hzau.edu.cn/bnapus/). This scaling up of available genome data has been mirrored by an extensive effort to predict resistance gene candidates, first in the single sequenced accession ‘Darmor-*bzh*’ with 425 NBS-LRR genes predicted in the first genome assembly (Chalhoub et al. 2014) and more recently based on a *B. napus* pangenome or on available Brassicaceae sequence data (Dolatabadian et al. 2020; Tirnaz et al. 2020). Dolatabadian et al. (2020) predicted 1,749 RGAs across 50 accessions of *B. napus*, including almost 400 absent from the reference genome.

Despite the huge numbers of *R*-genes predicted through in silico analyses in plants and *Brassica* spp. (Bayer et al. 2019; Dolatabadian et al. 2020), individual characterization of these candidates is still a major challenge and limiting factors exist in the use of this information for more applied genetics and breeding. Although such studies have provided useful and extensive databases for reference, the primary current use is likely to be in interrogating candidate genes identified to underlie experimentally determined resistance Quantitative Trait Loci (QTL) (Cantila et al. 2022), as merely the identification of a resistance gene candidate is not sufficient to know if this gene is in any way involved in any particular pathogen response. However, this method may increasingly gain utility as our understanding of gene and protein interactions, signaling and gene pathways improves, particularly in conjunction with transcriptomic approaches involved in the pathogen infection response (Borhan et al. 2022). A first approach developed to reduce the number of candidates is to compare predicted RGAs with previous knowledge obtained from the genetic mapping of resistance loci to identify robust candidates: such an approach has been carried out already in *B. oleracea* and *B. napus* (Bayer et al. 2019; Dolatabadian et al. 2020). Resistance gene analog prediction can also be compared or applied to transcriptomic data (e.g., Lowe et al. 2014; Becker et al. 2017, 2019; Hubbard et al. 2020; Song et al. 2021) and applied across accessions with known resistance responses and large databases such as BnPIR. This latter approach can be particularly useful, considering the high diversity in terms of gene content between accessions, to assess the conservation of a particular candidate resistance gene analog (Song et al. 2020).

### Validating resistance and avirulence genes

Despite the thousands of resistance gene analogs predicted using bioinformatic pipelines, only a limited number of *R* genes have been cloned, sequenced, and validated in plants (see Kourelis et al. 2018 for review). The first *R* gene to be cloned, in 1992, was *Hm1* in *Zea mays*, which encodes an enzyme involved in the detoxification of a toxin produced by the maize pathogen *Cochliobolus carbonum* (Johal and Briggs 1992).

Within the *B. napus-L. maculans* pathosystem, five *Brassica* resistance genes (i.e., *LepR3*, *Rlm2*, *Rlm4*, *Rlm7*, *Rlm9*; Larkan et al. 2013, 2015, 2020; Haddadi et al. 2022; Table 2) and 12 *L. maculans* avirulence genes have been cloned (i.e., *AvrLm1*, *AvrLm2*, *AvrLm3*, *AvrLm4-7*, *AvrLm5-9*, *AvrLm6, AvrLm10A*, *AvrLm10B*, *AvrLm11*, *AvrLm14*, *AvrLmS-Lep2*, *AvrLmSTEE98*; Fudal et al. 2007; Gout et al. 2006; Parlange et al. 2009; Balesdent et al. 2013; Ghanbarnia et al. 2015; 2018; Plissonneau et al. 2016; Petit-Houdenot et al. 2019; Degrave et al. 2021; Van de Wouw et al. 2014; Jiquel et al. 2021; Neik et al. 2022; Table 3). This number of avirulence genes cloned is higher than any other crop disease system so far, comparable to what is known in the *P. oryzae*-rice model (Hu et al. 2022), making the *Brassica-L. maculans* interaction a model system for the study of *R-Avr* gene interactions (reviewed by Rouxel and Balesdent 2017; Borhan et al. 2022). Before the availability of reference genomes and high-density genetic maps, identification of candidate genes underlying resistance loci from genetic mapping studies was a major challenge, involving now obsolete methods (at least in *Brassica*) such as chromosome walking (reviewed by Jander et al. 2002). The major methods still used for candidate gene identification and functional validation are often classified as “forward genetics” and “reverse genetics” methods. Forward genetics firstly involves inspection and functional annotation of candidate genes underlying mapped trait loci to predict which genes are most likely causal for the trait of interest: this can be facilitated by identifying the “type” of genes present and their predicted function (annotation). Subsequently, sequencing a subset of susceptible and resistant lines can determine if the two groups share different, putatively functional allelic variants of the same candidate gene, which is then likely to be directly related to the resistance phenotype. Reverse genetic approaches involve going from genotype to phenotype and include mutant screening methods such as TILLING (McCallum et al. 2000) where gene mutants are first identified before the phenotypic effect of the mutation is experimentally verified: reverse genetics approaches have to date not been used in the *Brassica*-*L. maculans* pathosystem, but may have potential in the future for validating candidate genes identified through genomic prediction methods.

**Table 3:** Avirulence genes in *L. maculans* and known *Rlm* gene interactions with *Brassica* species

*L. maculans AvrLm* genes	Cognate *Rlm* genes	Notes	*AvrLm* gene cloned	Reference
*AvrLm1*	*Rlm1* LepR3*	–	Yes	Gout et al. 2006
*AvrLm2*	Rlm2*	*Rlm2* and *LepR3* are alleles of the same gene	Yes	Ghanbarnia et al. 2015
*AvrLm3*	*Rlm3*	Presence of AvrLm4-7 masks interaction of Rlm3 and AvrLm3	Yes	Plissonneau et al. 2016
*AvrLm4-7*	Rlm4* Rlm7*	*Rlm4*, *Rlm7,* and *Rlm9* are different allelic versions of the same gene	Yes	Parlange et al. 2009
*AvrLm5-9*^a^	*Rlm5* Rlm9*	Presence of AvrLm4-7 masks interaction of Rlm9 and AvrLm5-9	Yes	Ghanbarnia et al. 2018 Ghanbarnia et al. 2018; Van De Wouw et al. 2014; Plissonneau et al. 2018
*AvrLm6*	*Rlm6*	–	Yes	Fudal et al. 2009
*AvrLm8*	*Rlm8*	–	No	Balesdent et al. 2002
*AvrLm10A*	*Rlm10*	*AvrLm10A* and *AvrLm10B* are both required to trigger resistance	Yes	Petit-Houdenot et al. 2019
*AvrLm10B*	*Rlm10*		Yes	
*AvrLm11*	*Rlm11*	–	Yes	Balesdent et al. 2013
*AvrLm13*^b^	*Rlm13*		No	Raman et al. 2021
*AvrLm14*	*Rlm14*		Yes	Degrave et al. 2021
*AvrLmS-Lep2*	*RlmS* *LepR2*	–	Yes	Yu et al. 2005; Neik et al. 2022
*AvrLep1*^c^	*LepR1*	–	No	Ghanbarnia et al. 2012; Yu et al. 2005
*AvrLep4*^*b*^	*LepR4*	–	No	Yu et al. 2013
*AvrLmSTEE98*^d^	*RlmSTEE98*		Yes	Jiquel et al. 2021

^*^indicates cloned *Rlm* genes

^a^*AvrLm5-9* was first named *AvrLmJ1* (van de Wouw et al. 2014) and then identified as *AvrLm5* by Plissonneau et al. (2018). Finally, Ghanbarnia et al. (2018) identified the double specificity toward *Rlm5* and *Rlm9*,

^b^This avirulence gene is only postulated to date since it has not been genetically identified or mapped,

^c^This gene has been genetically identified and mapped, but not cloned,

^d^*AvrLmSTEE98* encodes for an avirulence protein recognized during stem colonization

### Cloning of avirulence genes

Forward genetic approaches have been very successful in cloning avirulence genes in the *L. maculans*/*B. napus* pathosystem, with *AvrLm1*, the first *AvrLm* gene cloned in 2006 (Gout et al. 2006). This gene was previously genetically identified by Ansan-Melayah et al. (1995) using a map-based cloning strategy which allowed the identification of the genetic interval on a Bacterial Artificial Chromosome (BAC) contig. This cloning highlighted interesting characteristics of *AvrLm1* and its genomic environment: located in the middle of a large TE-rich region, *AvrLm1* itself encodes a small protein (205 amino-acids) with one cysteine residue and a peptide signal and has no homology with other sequences available in public databases. It was later shown that these characteristics (except for cysteine enrichment) were shared by all avirulence genes that were subsequently cloned (subsequently referred to as SSPs for Small Secreted Proteins), together with low or no expression during axenic growth and specific induction during the asymptomatic infection of *B. napus*, at the beginning of the infection period (except for *AvrLmSTEE98*; Rouxel et al. 2011; Jiquel et al. 2021). *AvrLm1*, *AvrLm6,* and *AvrLm4-7* were all cloned using the same strategy (Gout et al. 2006; Fudal et al. 2007; Parlange et al. 2009) before the release of the *L. maculans* genome (Rouxel et al. 2011). Since then, availability of the genomic sequence of *L. maculans* has facilitated cloning of additional avirulence genes: an exception was *AvrLm3* which required a combination of several strategies for successful cloning (map-based cloning, RNA-seq analysis, BAC sequencing; Plissonneau et al. 2016), most likely because it is located in a telomeric region, surrounded by repeats and absent from the initial reference genome assembly of *L. maculans*. Cloning of *AvrLmS-Lep2* was undertaken using two independent approaches: a typical map-based cloning approach and bulked segregant sequencing of progeny produced by crossing between virulent and avirulent isolates (Neik et al. 2022). Avirulence genes *AvrLep2* and *AvrLmS* were previously shown to trigger resistance in cultivar ‘Surpass400’ of *B. napus* which contains *RlmS* while other sylvestris-derived cultivars contain *LepR2*. Cloning suggested that these are the same gene, which was therefore named *AvrLmS-Lep2*.

All avirulence genes cloned to date are initially overexpressed following the infection of oilseed rape, where interaction between an *AvrLm* gene and its cognate *Rlm* gene triggers a resistance response at the onset of the infection. Nevertheless, the recent characterization of the *AvrLmSTEE98/RlmSTEE98* interaction highlighted that a gene-for-gene interaction could be involved in the limitation of stem colonization and triggering of partial resistance phenotypes (Jiquel et al. 2021). Examples such as these will undoubtedly help rethink our current categories of “qualitative” and “quantitative” resistances in the *L. maculans*/*Brassica* pathosystem and open the way to identification of further gene-for-gene interactions expressed at other plant growth stages, and/or on other plant organs than previous gene-for-gene interactions uncovered so far in this pathosystem at the cotyledon/leaf stage.

### Cloning of resistance genes

Although the first *AvrLm* gene was cloned in 2006, it was only in 2013 that the first major resistance gene in *B. napus* against stem canker was cloned (*LepR3*; Larkan et al. 2013). Cloning and characterizing *Rlm* and *Avrlm* genes generally follows a standard approach: firstly, identification of genotypes with opposite phenotypic characteristics (susceptible and resistant lines for the plant species; avirulent and virulent isolates for the fungus), followed by one, or several, cross(es) between these genotypes, then genotypic and phenotypic analysis of resulting segregating progeny to identify genomic regions associated with the resistance phenotype. Distribution of the resistance/virulence phenotype across phenotypic categories as well as Mendelian segregation ratios can help in determining if one or more genes/loci are responsible for the trait. Depending on the size of the resulting associated region/s, candidate genes may then also be investigated within these regions, usually firstly by functional annotation (gene ontology predictions based on sequence comparisons to genes of known function), then by validation of gene function. The most common method of functional validation involves the transformation (cloning and insertion) of the candidate resistance gene into a susceptible background and screening of the transformants for resistance.

*LepR3* was mapped on chromosome A10 in 2008 (Yu et al. 2008); then, cloning from the cultivar ‘Surpass400’ (Li and Cowling 2003; Li et al. 2007) was finally achieved in 2013. To achieve cloning of *LepR3*, the authors developed an elegant approach to identify candidate genes, with the improvement in the genetic map to provide a high-resolution mapping of *LepR3* coupled with the use of data from closely related species *B. rapa* and *A. thaliana* to investigate collinearity between species for the *LepR3* region (Larkan et al. 2013). The *B. rapa* region containing the *LepR3* genetic interval was then annotated, and subsequent sequencing of the corresponding locus from *B. napus* allowed for the identification of a candidate gene encoding a Receptor-Like Protein (RLP). The parental genotypes both had the genetic locus, but the resistant cultivar ‘Surpass400’ had a longer coding sequence for the gene than the susceptible cultivar ‘Topas DH16516’, as well as sequence variations. *LepR3* triggers a hypersensitive response during interaction with *AvrLm1*-isolates of *L. maculans,* which is also involved in a gene-for-gene interaction with *Rlm1* (Larkan et al. 2013; Table 3). Since the cloning of *LepR3* by Larkan and colleagues, the same authors successfully cloned four other *Rlm* genes (*Rlm2*, *Rlm4*, *Rlm7,* and *Rlm9*; Larkan et al. 2015, 2020; Haddadi et al. 2022). The second *Rlm* gene to be cloned was *Rlm2* (Larkan et al. 2015), inducing a hypersensitive response during interaction with *AvrLm2*-isolates of *L. maculans* (Table 3). It was previously shown that *Rlm2* and *LepR3* were located in the same genetic interval (Larkan et al. 2014) and amplification of the candidate gene before transformation of a susceptible genotype for gene functional validation purposes was performed using the same primers as for amplification of *LepR3*. The authors have shown that *LepR3* and *Rlm2* are alleles of the same gene, yet trigger a hypersensitive response during interaction with isolates containing *AvrLm1* or *AvrLm2*, respectively. Besides these two RLP-encoding genes, *Rlm9*, the third cloned *Rlm* gene, is a wall-associated kinase-like (WAKL) encoding gene (Larkan et al. 2020). The genetic interval was previously identified on chromosome A07 of the cultivar ‘Darmor-bzh’ (Larkan et al. 2016a; Table 2) and forms a genetic cluster with *Rlm4*, *Rlm3,* and *Rlm7*. Haddadi et al. (2022) recently showed that *Rlm4* and *Rlm7* are alleles of *Rlm9*, and it is postulated that *Rlm3* is yet another allele of the same gene. Cloning of *Rlm* genes demonstrated that different *Rlm* genes can correspond to alleles of the same gene, or might correspond to the same gene and that different resistance genes can trigger resistance via interaction with the same *AvrLm* gene (for instance, both *LepR3* and *Rlm1* trigger resistance toward *AvrLm1*; Table 3). Larkan et al. (2013) and Rouxel and Balesdent (2013) postulated that *Rlm1* and *LepR3* could be the same gene, although they occupy different genomic loci, with *Rlm1* on chromosome A07 and *LepR3* on chromosome A10. A translocation of *Rlm1* could have given rise to *LepR3* in ‘Surpass400’ (Van De Wouw et al. 2009). Despite the cloning of *AvrLm* and *Rlm* genes, much still needs to be elucidated given the complexity of the pathosystem, and more and more complex situations have been uncovered as the number of cloned genes has increased.

In contrast with many other pathosystems in which NLR intracellular receptors are the rule, cloned *Rlm* genes mostly correspond to cell surface receptor-encoding genes. So far, *Rlm9* and *Stb6* (a resistance gene operating in wheat against *Z. tritici*) are the only WAKL resistance genes to be cloned (Saintenac et al. 2018). This indicates that RLP or RLK types are not only associated with PAMP-triggered immunity, but may also be main players in effector-triggered immunity against apoplastic pathogens such as *Z. tritici*, *L. maculans,* or *Fulvia fulvum* (for review on *Cladosporium fulvum* resistance genes; de Wit 2016; Zhao et al. 2022).

### Expression of *Rlm* and effector (including *AvrLm*) genes

Cloning of *AvrLm* and *Rlm* genes together with the development of more and more advanced gene expression analysis techniques has allowed a deeper understanding of their expression during the whole infection cycle, including spatial and temporal regulation. At first, analysis of *AvrLm* gene expression was done upon cloning using qRT-PCR, then a broader analysis of gene expression was made possible using microarray technology following the publication of the genome sequence of *L. maculans*, and most recently, RNA-seq analysis has allowed an in-depth exploration of the transcriptome (Gay et al. 2021). Accumulation of these data and the increase in data quality has deepened our understanding of the complexities of effector gene expression. RT-PCR analysis of *AvrLm1* expression showed an upregulation of this gene 7 to 12 days post-infection (Gout et al. 2006). Microarray analysis highlighted coordinated expression of a set of effector genes, including all cloned *AvrLm* genes together with other effector candidates, with low (or no) expression during axenic growth and a strong over-expression during the asymptomatic infection of *B. napus*. Specifically, *AvrLm* genes and other effector candidate genes showed a peak of expression seven days post-inoculation, before the appearance of the first visible symptoms on cotyledons (Rouxel et al. 2011). A better knowledge of effector gene expression was rendered possible with the first RNA-seq analyses performed at different stages of the interaction between *L. maculans* and *B. napus* (Gervais et al. 2017). The authors performed RNA-seq analysis on cotyledons and stem, showing that a subset of candidate effector genes were expressed specifically during stem colonization and suggesting a possible role of these effectors in necrosis establishment at this later stage of the lifecycle. These were renamed ‘late’ effector genes to distinguish them from *AvrLm* genes specifically induced during primary asymptomatic infection of *B. napus*. Analysis of a later infection stage and a different plant organ than cotyledons (from which most transcriptomic data were generated so far) revealed a more complex expression of effector genes and suggested different roles of subsets of effectors that are expressed at distinct life stages. Gene expression analysis throughout the life cycle of *L. maculans* (Gay et al. 2021) revealed a very complex regulation of the genes involved in pathogenesis, far more sophisticated than initially postulated from analyses carried out during early infection of cotyledons or petioles under controlled conditions (Rouxel et al. 2011; Gervais et al. 2017; Gay et al. 2021). Based on previous analyses of *AvrLm* gene expression from microarray data (Rouxel et al. 2011) or from the first RNA-seq analysis performed on stems (Gervais et al. 2017), the situation remained quite simple, with a subset of effectors, the *AvrLm* genes, expressed solely during primary infection. This suggested the involvement of these genes in manipulating the host immune defense system to ensure establishment of the infection. The thorough analysis of the *L. maculans* transcriptome throughout the life cycle, under controlled conditions, and in the field, in different plant organs (e.g., cotyledons, leaf, stem, residues), covering different life stages of the fungus (e.g., mycelium, ascospores) and all lifestyles displayed by the fungus (asymptomatic, necrotrophic, endophytic, saprophytic) compared to axenic culture (on several media) has deepened our understanding of the complexities of (candidate) effector gene expression. RNA-seq data showed that eight specific clusters of genes, all enriched in effector genes, were expressed during interaction with oilseed rape and specifically associated with a given lifestyle and/or an infected tissue. Gay et al. (2021) showed that expression of *AvrLm* genes was finely regulated such that activation occurs exclusively during the different asymptomatic colonization stages, not only during primary infection of cotyledons or leaves.

In contrast with effector genes, much is yet to be discovered regarding *Rlm* gene expression. Becker et al. (2017) performed RNA-seq and gene expression analysis using qRT-PCR following laser microdissection to provide a thorough analysis of the global response of a resistant and a susceptible genotype of *B. napus* together with a specific analysis of the infection site. Differentially expressed genes underlying the infection response in resistant genotypes were enriched with genes involved in pathogen recognition, cell signaling, and vesicular trafficking, and included genes encoding NBS-LRR receptors, wall-associated kinases, RLKs and RLPs (Becker et al. 2017). The recent cloning of *Rlm* genes also made it possible to investigate their expression during infection. For instance, *Rlm9* was specifically up-regulated in ‘Darmor’ (containing *Rlm9*) compared to a susceptible genotype following infection with an *AvrLm5-9* isolate (Larkan et al. 2020). Moreover, *Rlm9* shows a temporal expression pattern, with a low induction at three days post-inoculation, a peak of expression at six days post-inoculation, and a slight decrease in expression up to nine days post-inoculation. This pattern resembles that of the *AvrLm* genes cloned so far.

### Complex interactions between *Rlm* genes and *AvrLm* genes

To date, *L. maculans-Brassica* spp. is the pathosystem for which the largest number of avirulence genes have been cloned, along with several of the corresponding plant resistance genes. This has highlighted that interactions between avirulence proteins and their cognate R proteins do not always follow the classic gene-for-gene interaction model, with more and more peculiar cases uncovered as new avirulence genes are cloned (such as two-for-one models, different allelic interactions, and epistatic suppression of phenotypes).

As previously mentioned, an important characteristic of the gene-for-gene model is the discovery of increasing complexity, invalidating the assumption that one gene is always related to another single gene. Several discoveries of such complex interactions have been recorded, such as *AvrLm1*, which is recognized by *LepR3* and *Rlm1* (Larkan et al. 2013; Table 3). Another example is the two-gene for one-gene interaction between both *AvrLm10A* and *AvrLm10B* and *Rlm10*. These two avirulence genes are necessary to induce avirulence toward *Rlm10* (Petit-Houdenot et al. 2019; Talbi et al. 2023). Some other avirulence genes have dual recognition specificities, such as *AvrLm4*, which was renamed *AvrLm4-7* as it can generate a resistance response in the presence of both *Rlm4* and *Rlm7* (Parlange et al. 2009), or *AvrLm5-9* that induces responses to both *Rlm5* and *Rlm9* (Van De Wouw et al. 2014; Ghanbarnia et al. 2018;). The fourth series of examples illustrate the “camouflage model” whereby one avirulence may mask the recognition of another avirulence by the matching resistance. There are two examples here. Firstly, *AvrLm4-7* hides the presence of the *AvrLm3* isolate and prevents recognition by Rlm3, even when *AvrLm3* is present and expressed (Plissonneau et al. 2016). Deletions or inactivating mutations of *AvrLm4-7* lead to unmasking and recognition of AvrLm3, while other mutations such as those generating virulent isoforms of the AvrLm3 protein, or isolates that contain point mutations in *AvrLm4-7*, escape Rlm7 resistance while maintaining the suppression of the AvrLm3 phenotype (Plissonneau et al. 2017; Balesdent et al. 2022). These studies show that the *AvrLm3* gene, once thought to be lost due to the high selection pressure caused by widespread *Rlm3*-containing cultivars, is still present and expressed (Rouxel and Balesdent 2017). A second example is the *AvrLm5-9* host recognition, which is also masked by the presence of *AvrLm4-7*. The presence of *AvrLm4-7* masks the recognition of AvrLm5-9 by Rlm9, similar to what is known for the *AvrLm4-7* masking of AvrLm3. AvrLm5-9 and AvrLm4-7 do not interact, and the presence of *AvrLm4-7* does not suppress the expression of *AvrLm5-9*.

## A history of resistance gene use and the evolution of populations on different continents

### Use of resistance genes in Europe, Canada and Australia

As mentioned previously, breeding for resistance in *B. napus* was initially based on mass selection, picking up the more resistant plants at the end of the growing season for further breeding, under the assumption that the resistance was under quantitative genetic control (Cowling 2007). These approaches inadvertently identified and selected major gene resistances, which were the most efficient genetic factors for control of the disease in a context where the dominant race of the fungus was locally avirulent. The increasing popularity of inbreeding to generate pure, homogenous lines also contributed to the impoverishment of *Rlm* gene diversity present in the initial varieties (see the example of ‘Tina’ or ‘Quinta’ above), eventually generating cultivars harboring a single resistance gene. In many cases retrospective analysis using genetically bred isolates allowed identification of the resistance genes used in early breeding strategies, whenever the genotypes were still available in collections.

Europe, being the first to breed for improved genotypes with high agronomic value, and being faced early with the stem canker problem, was also the first to breed (unknowingly) for major *Rlm* genes (Rouxel and Balesdent 2017). In France, for example, *Rlm2* was released in commercial cultivars in 1970, *Rlm4* in 1971 (with extreme commercial success at the European scale when introduced in cv. ‘Jet Neuf’ in 1977), *Rlm1* in 1992 (with extreme commercial success when introduced in cv. ‘Capitol’ in 1995), and *Rlm7* in 2002 (with an extreme commercial success when introduced in cultivars such as ‘Exocet’ and ‘Exagone’ from 2004 on) (for review see Rouxel and Balesdent 2017). In addition, *Rlm3* and *Rlm9* have been present for years in cultivars, but there is no definite information on when and in which cultivars they were first released (e.g., for *Rlm3* in Balesdent et al. 2022), and without displaying any efficient resistance toward populations of the fungus that were 100% virulent at the European scale (Stachowiak et al. 2006). More recently, two *sylvestris*-derived genes were introduced in winter type European cultivars: *RlmS-LepR2* in 2016 (Balesdent et al. 2024) and *LepR1* in 2021 (M.-H. Balesdent and A. Jiquel, unpublished data). It has also to be mentioned that *Rlm1*, *Rlm2*, *Rlm3*, *Rlm4,* and *Rlm9* have been (or are still) maintained in cultivars long after they have been defeated (Balesdent et al. 2022; M.-H. Balesdent, unpublished data).

In Australia, the first resistance gene used was *Rlm4*, in 1974 (Roy and Reeves 1975; Rouxel et al. 2003b) followed in 2000 by the use of the *sylvestris*-derived resistance (*RlmS-LepR2* and *LepR3—*firstly identified as *Rlm1* by Van De Wouw et al. (2009)). From the late 1990s onwards, Australian canola breeders began to incorporate European winter germplasm, especially from France, more extensively into their breeding programs, allowing diversification of the *Rlm* gene complement (Marcroft et al. 2012). In an extensive screening of 127 Australian cultivars and advanced breeding lines done in the early 2010s, 29% contained *Rlm4*, 27% contained *Rlm1* (or *LepR3*), 21% contained *Rlm9*, 10% contained *RlmS*-*LepR2*, 9% contained *Rlm3*, and 5% contained *Rlm2* (Marcroft et al. 2012). More recent data suggest *Rlm6*, introgressed from *B. juncea*, is also available in some Australian cultivars (Van de Wouw and Howlett 2020). However, the use of specific resistance genes in Australia is sometimes unclear due to variable naming conventions and changes in gene content in the same genotype from one paper to the other. Mainly, the *sylvestris*-derived resistance genes in ‘Surpass400’ were first identified as *Rlm1* and *RlmS* (Van De Wouw et al. 2009), with confusion between *LepR3* and *Rlm1* (both recognizing *AvrLm1*), while *RlmS* was later identified as *LepR2* and renamed *RlmS-LepR2* (Neik et al. 2022). Also, *Rlm5* (from *B. juncea*) is suggested to be absent from Australian genotypes but corresponds to one resistance group in the Australian classification of resistant cultivars (Van de Wouw et al. 2017; Van de Wouw and Howlett 2020). In Australia, there are strong incentives to rotate resistance genes as much as possible to alternate selection pressures on the pathogen, and only local breakdowns of resistance have been documented, likely due to the lack of cropping of one single resistance gene on large acreages for extended periods, in contrast with what is observed in Europe.

In Canada, the first documented *Rlm* gene used was *Rlm3*, released in 1995. *Rlm3* was then used in numerous varieties for years and was present in up to 55% of the registered varieties in the mid-2010s (Zhang et al. 2016). While *LepR3/Rlm1*, *Rlm2*, *Rlm3*, *Rlm4*, *Rlm9*, *RlmS-LepR2,* and *LepR1* have been documented to be present in Canadian genotypes, at present, only four *Rlm* genes are commercially available in Canadian varieties: *LepR3/Rlm1*, *Rlm3*, *Rlm4* and *RlmS-LepR2* (Zhang et al. 2016; Cornelsen et al. 2021).

### The durability of resistance in the *L. maculans*/*B. napus* pathosystem

The wide use of major resistance genes in crops is famed for rapidly selecting populations of the pathogen that have become virulent. The speed of this “breakdown” is theoretically linked to the “evolutionary potential” of the pathogen (McDonald and Linde 2002), but also depends on more complex traits including the fitness cost for the pathogen to lose the AvrLm protein effector function, the complexity of the gene-for-gene interaction, and the pre-existence of virulent isolates in the populations. *L. maculans* sums up a series of biological and epidemiological traits that allows it to “breakdown” in a few years only, novel resistance genes deployed over large areas due to their commercial success. These include a mixed reproduction regime and an obligate annual sexual reproduction favoring recombination of avirulence loci, very high local population size (ascospores resulting from sexual reproduction and produced for most of the vegetative life of rapeseed), and location of avirulence genes in plastic regions of the genome which undergo accelerated mutation rates. Thus, the generation of novel virulent isolates which can break down novel resistance genes is mostly a local and self-sustaining process, and virulence generated at each sexual cycle is selected and amplified in populations whenever a new resistance is used (Rouxel and Balesdent 2017).

Historically, in Europe, *Rlm* gene use has led to the breakdown of resistance at the country scale for *Rlm2* (in 1972 in France), *Rlm1* (in 1999 in France), *Rlm4* (possibly at the end of the 1990s), and *Rlm7* (in 2022 in France) (Rouxel and Balesdent 2017; Balesdent et al. 2022). The absence of virulent isolates at the *AvrLm2* and *AvrLm9* loci in Europe may also indicate an ancient, undocumented breakdown of these two resistance genes. Another well-documented breakdown event is that of the *sylvestris* resistances (*LepR3* and *RlmS-LepR2*), present in ‘Surpass400’, in the Eyre Peninsula (Australia) in 2003 and then in other parts of Australia (Sprague et al. 2006). Finally, Western Canada experienced an *Rlm3* breakdown between 1997 and 2010 (Zhang and Fernando 2018). The speed of these different breakdown events differed between the different situations, and this evaluation may depend on the definition given by the authors of “breakdown”: in other words, when do we consider that a resistance gene has “broken down”? This may depend on the geographic scale considered, the ratio of virulent to avirulent isolates in the population, the amount of yield losses, or other factors. Thus, the literature documents very rapid breakdowns, e.g., the 5-year breakdown of *Rlm1* at the French scale (with more than 90% of the isolates in populations having shifted to *AvrLm1* virulence) or of the 3-year breakdown of *LepR3* (also favoring the evolution of isolates virulent at the *AvrLm1* locus) in the Eyre Peninsula (Australia) (Rouxel et al. 2003a; Sprague et al. 2006). It is also probable that the breakdown of *Rlm2* in France has been a very rapid event with only two years between the release of a successful *Rlm2* cultivar and documented susceptibility of the cultivar (Rouxel and Balesdent 2017). However, for reasons which are still mostly elusive, and may differ between regions, plant genotypes, cropping practices, and complex interactions between *AvrLm* genes and resistance genes themselves, such rapid breakdowns are not always the rule. For example, the breakdown of *Rlm3* in Canada was more a slow erosion of the efficiency of the gene over decades than a rapid breakdown, even though *Rlm3* was widely used in Canadian varieties (Zhang and Fernando 2018). Similarly, and contrasting with what has been observed in experimental settings (Daverdin et al. 2012), the breakdown of *Rlm7* in France, massively deployed since 2004, has been a rather slow process (Balesdent et al. 2022). All of this indicates that a priori postulation on the durability of an *Rlm* gene is elusive, and necessitates knowledge of the importance of the *AvrLm* gene for the fungus, its mutability, and the existence of epistatic effects masking other avirulence genes (Balesdent et al. 2022).

The durability of the resistance must also consider the possible pre-existence of virulent isolates in populations. While it is now well established in Europe that virulent isolates for *AvrLm1*, *AvrLm7,* or *AvrLmS-Lep2* loci were extremely rare at the time of introduction of the corresponding resistance gene (Rouxel et al. 2003a; Balesdent et al. 2022, 2024), this may not be the case in Australia or Canada, reinforcing the ease with which new resistances are broken down. For example, a survey done in Western Canada by Dilmaghani et al. (2009) in 2004–2006 indicated that virulence at *AvrLm1*, *AvrLm4*, *AvrLm7,* or *AvrLm9* loci predated the use of the resistance in commercial varieties and that extreme diversity exists even between different locations in the same province. In Manitoba (Canada), some locations have 100% of isolates virulent at the *AvrLm1* locus, while others have up to 62% avirulent isolates. Similar disparities were found for *AvrLm4* or *AvrLm7*, with some locations harboring 100% virulent isolates, while others have up to 75% avirulent isolates (Dilmaghani et al. 2009). Similar findings were obtained by Cornelsen et al. (2021) with a high number of isolates virulent at the *AvrLmS*-*Lep2* locus, suggesting virulent isolates were widely present in the population before the use of the corresponding *Rlm* gene in commercial varieties. While such extensive surveys have not been performed in Australia, a retrospective examination of a collection of 26 isolates (including two from New Zealand) collected between 1971 and 2002 by Balesdent et al. (2005) indicated that 77% of the isolates were virulent toward *Rlm4*, consistent with the use of this resistance gene in Australian varieties, but also that 54% were virulent at the *AvrLm1* locus and 65% at the *AvrLm7* locus. This pre-existence of virulent isolates may have aided the rapid breakdown of the ‘Surpass400’ resistance, as already postulated by Sprague et al. (2006), and, more recently, aided the breakdown of *Rlm7* in two fields in Victoria (Van de Wouw et al. 2022). These data point to another major difference between Europe and Australia/Canada: while in Europe, successive use of *Rlm* genes had a strong effect on fungal population structure, eliminating one by one avirulence at loci *AvrLm1*, *AvrLm2*, *AvrLm4*, *AvrLm7* and *AvrLm9* from the populations, the situation is more complex for Australia and Western Canada, with a high diversity of races, often at the local scale, independent of the use of *Rlm* genes, and with virulent races predating the use of the corresponding *Rlm* genes.

### Population surveys to aid durable management of resistance

To maximize the durability of cultivars with new resistance, precise information on race structure is needed. This could be achieved by scouting the races present in the field at different scales prior to and during the use of these cultivars, leading to the development of strategies to maximize the durability of a rare resource. To avoid the use of tedious and space-consuming pathogenicity tests, molecular markers for each *AvrLm* gene are tools of choice to speed up population characterization and improve screening throughput. The design of such markers requires that the molecular events leading to virulence are extensively characterized and are followed up on to track down the dynamics of events that lead to a fixed virulent allele (Rouxel and Balesdent 2017; Gautier et al. 2023). Unfortunately, there may be only limited information for some of the genes at present in the literature, and over-simplification of available data may lead to inaccurate recommendations for the best markers to use. In general, when the fungal populations are challenged by a new resistance, a common immediate response is the elimination of the *AvrLm* gene via drastic events (Gout et al. 2007; Daverdin et al. 2012; Fudal et al. 2009). This is facilitated by the hosting of these genes in large TE-rich regions of the genome, and obligate reproduction favoring repeat-induced point mutation (RIP) events at each sexual cycle. As a consequence, the first step to virulence is either complete deletion or mutation to inactivation via RIP (Rouxel and Balesdent 2017). In France, this was observed for *AvrLm1* and *AvrLm7* in agricultural settings (Rouxel and Balesdent 2013; Balesdent et al. 2022) and for *AvrLm6* and *AvrLm7* in experimental fields (Fudal et al. 2009; Daverdin et al. 2012). However, even when hosted in TE-rich regions, including subtelomeric regions, some genes (such as *AvrLm2*, *AvrLm3,* or *AvrLm9*) are never deleted or inactivated, and a virulent allele has formed by point mutation (or mutations) and replaced the avirulent form in populations (except when remaining masked via epistatic interactions as found for *AvrLm3*; see below). In contrast, another gene, *AvrLm11*, is spontaneously lost due to being located in a dispensable chromosome of *L. maculans*, even in the absence of selection pressure (Balesdent et al. 2013). For currently used resistance genes in Europe, three cases can thus be distinguished. The first is where *AvrLm* genes corresponding to *Rlm* genes have been present in genotypes for ages and have led to fixed alleles, probably following a first round of drastic events such as deletions. There are different cases here: genes that are deleted and never recovered (*AvrLm1* being the sole example known) and genes in which the avirulent allele has been replaced by a virulent one, resulting in a single amino acid substitution (as in *AvrLm2*, *AvrLm4*, and *AvrLm9*). The second case is where *AvrLm* genes correspond to *Rlm* genes that have never been used in commercial varieties, and for which there is only limited information on molecular evolution toward virulence in agricultural settings. Thirdly, *AvrLm* genes may also be masked from recognition by the matching *Rlm* gene due to epistatic interactions between *AvrLm* genes. In this respect, a recent comprehensive study has been performed by Gautier et al. (2023) that identified numerous alleles at each avirulence locus, and showed that the diversity of alleles found in a population is gene dependent and independent of the selection pressure to which the population has been submitted. This work is currently complemented by a large international effort to gather and sequence numerous *L. maculans* isolates from all parts of the world and to capture their diversity at avirulence loci (van de Wouw et al. 2024). These genomic factors driving rapid diversification at *AvrLm* loci need to be considered when deploying new resistance genes, and extensive data on sequence variation at avirulence/virulence loci will be instrumental to designing molecular markers to survey populations of the pathogen.

## Sustainable management: breeding strategies to build genotypes with durable resistance

Breeding for resistant cultivars of *B. napus* is widely agreed to be one of the best management strategies for ensuring crop production while under pressure from the *L. maculans* pathogen (reviewed by Borhan et al. 2022). However, ensuring the durability of the genetic resistance in the crop cultivars can be challenging. The fungal pathogen is to date able to breakdown all resistances known in *B. napus*, and genetic resources which would enable further diversification of *Rlm* genes within commercial cultivars are tapped out within the primary *B. napus* germplasm pool. From this perspective, sustainable use and rotation of existing resistances within the crop (Van de Wouw et al. 2021) coupled with pathogen-informed management strategies (reviewed by Li et al. 2021) are critical strategies for sustaining yield in *Brassica napus*. To effectively match resistance in the cultivars with existing isolate populations, a great deal of information is required: the exact *Rlm* gene composition of the cultivars involved, the *AvrLm* gene content of the pathogen populations at the cropping location, and any possible effects of gene interactions, environmental effects, and cultivar genetic backgrounds on the Rlm-AvrLm interaction in the field. Despite the complexity, this is still an achievable strategy, particularly with recent advances in high-throughput genotyping and our understanding of this crop-pathogen interaction (Borhan et al. 2022).

### A critical discussion of the pyramiding strategy

A major strategy which may help build durable resistances into cultivars is that of gene pyramiding (Stam and McDonald 2018). In this strategy, cultivars are developed to contain multiple resistance genes, allowing for broad-spectrum resistance against a range of isolate populations. In a broader sense, pyramiding may be the combination of major gene resistance with quantitative resistance within a single cultivar. This specific strategy is likely to offer the best protection against both major gene breakdown and the possibility that the fungus will evolve under pressure to simultaneously overcome multiple major resistance genes (Leflon et al. 2007; Pilet-Nayel et al. 2017). In all cases, pyramiding of exclusively major resistance genes in a single genotype has to be carefully thought over, keeping in mind possible adverse effects and the possibility of facilitating resistance breakdown across multiple genes. This was illustrated by the combination of *Rlm3-Rlm7* in current French varieties that favored a rapid adaptation in the fungus to generate double-virulent isolates with a single mutation in the avirulence gene (Balesdent et al. 2022), an outcome that should be avoided at all costs. This points again to the importance of preliminary dissection of the types of interactions that may exist between avirulence genes.

Combining major gene resistance and quantitative resistance may also be insufficient. In Australia from 1970 to 2000 for instance, breeding for polygenic blackleg resistance was possible thanks to diverse ancestral parents, but by 2000 there were signs of loss of genetic diversity and genetic drift leading to the breakdown of the quantitative resistance background (Cowling 2007). Combining quantitative and major gene/qualitative resistance sources may, however, still offer a viable breeding strategy for the future, particularly when coupled with a better understanding of quantitative resistances and the development of molecular markers which can help to accurately detect QTL and integrate them into breeding programs (Pilet-Nayel et al. 2017). In particular, the recent finding that some adult-stage resistances may be under simple genetic control (Jiquel et al. 2021, 2022) may favor the generation of markers to easily construct genotypes harboring both major gene resistance (operating at the leaf level) combined with a series of single gene quantitative resistances (operating during stem colonization).

### Resistance genes in related species: an opportunity to be seized

One of the greatest challenges for the future will be the diversification of the available *R* gene pool. This is a critical point to design strategies to ensure durable use of the few resistance sources available and to avoid sequential marketing of one gene after the other in a way that facilitates breakdown and has been the rule in Europe since the early 1970s. To achieve this goal in inbred *B. napus*, it will be necessary to look to related species (Katche et al. 2019). *Brassica napus* germplasm can be readily enriched by using resynthesis: genotypes of progenitor species *B. rapa* × *B. oleracea* can be utilized to directly produce 2*n* = AACC types, which are optimal for further breeding (Katche et al. 2023). Direct crossing between *B. rapa* and *B. napus* can also be carried out, followed by selection for euploid (2*n* = AACC) progeny, likewise crosses between *B. oleracea* and *B. napus,* although these often require embryo rescue (reviewed by FitzJohn et al. 2007). All crosses involving non-adapted germplasm can, however, be challenging for breeding programs: wild relatives generally carry undesirable agronomic traits, which complicates the use of this material for breeding purposes (Seyis et al. 2003). However, one successful example of the use of resynthesized *B. napus* is the cultivar ‘Varola 50 syn Surpass 400’ (Anon 2001), which contains many resistance genes coming from the A genome of *B. rapa* (Table 1).

The *Brassica* B genome is a particularly good source of stem canker resistance genes for breeding purposes (Navabi et al. 2013), but one which is difficult to use for rapeseed crop improvement. Resistance genes found in *Brassica* A or C genomes of related species can be transferred directly into *B. napus* in interspecific hybrids via homologous recombination, but B genome resistance genes rely on the much rarer recombination events between divergent genomes (homoeologous—ancestrally homologous) to transfer resistance genes (Chèvre et al. 2018). Previous attempts to introgress B chromosomes from *B. carinata* into *B. napus* to transfer stem canker disease resistance were not successful, but B genome chromosomes were inherited as whole linkage groups and some were retained in further generations (Navabi et al. 2011). Dhaliwal et al. (2017) identified B genome introgressions from *B. carinata* into *B. napus*, but stem canker disease resistance was not assessed in this study (the trait of interest was pod-shatter resistance). Dixelius and Wahlberg (1999) studied the conservation of specific regions of the B genome from *B. nigra*, *B. juncea,* and *B. carinata* in a BC_3_ population backcrossed to *B. napus*, but found no B genome chromosomes or chromosome arms. However, a few successful introgressions of resistance into *B. napus* have been recorded, such as the case of the introgression of the *Rlm10* gene on chromosome B4 of *B. nigra* (Chevre et al. 1996) or the transfer of *Rlm6* from *B. juncea* to *B. napus* (Balesdent et al. 2002).

### Structural families of *AvrLm*/effector proteins

In addition to these somewhat “classical” approaches to increase the number of available resistances, new prospects may arise through molecular engineering of selected plant immune receptors. This strategy is based on the new finding that fungal effectors, despite showing little or no sequence similarity, may belong to structural families (Outram et al. 2022). Structural families may be found in the same fungal species (like the MAX effectors in *Magnaporthe*, or the LARS family in *L. maculans*; Lazar et al. 2022), and thus may be recognized by closely related resistance proteins that may ideally be engineered to increase their recognition specificities (Cesari et al. 2022). Interestingly, structural families may also be common to many fungal genera, providing the potential to engineer multispecies recognition and resistance. This is, for example, the case for the LARS effector family of *L. maculans* that encompasses AvrLm3, AvrLm4-7, and AvrLm5-9, but also the Ecp11-1 effector of the unrelated fungal species *F. fulvum* (Lazar et al. 2022). The recent release and improvements in the deep learning neural network-based prediction software AlphaFold (Jumper et al. 2021) have already allowed structural prediction for thousands of fungal effectors (Seong and Krasileva 2023), heralding a new era in understanding effector recognition and making steps toward in silico engineering of resistance genes (Outram et al. 2022).

## Conclusion

Breeding for stem canker disease resistance is important, as it is one of the major diseases of rapeseed/canola worldwide. Major strategies currently in place to improve stem canker resistance in rapeseed are (1) identification and exploitation of genes present in different commercial *B. napus* cultivars for crop rotation and management purposes; (2) identification and utilization of quantitative genetic resistance, preferably also via pyramiding with existing major gene loci; and (3) identification of novel sources of stem canker resistance in diverse germplasm and wild relatives, and subsequent transfer to the cultivated crop gene pools. Here, we have documented progress to date in identifying resistance genes and resistance sources in rapeseed and avirulence genes in blackleg; most of the work to date has involved classical genetic mapping followed by sequence- or transformation-based gene validation/reverse genetics approaches. In the future, the development of molecular engineering approaches based on structural predictions of resistance proteins and further advances in bioinformatics and biotechnology may also substantially contribute to the production of resistant lines. Supervised machine learning approaches have already been applied for many years to predict R genes in plants and secreted effector proteins produced by plant pathogens (reviewed by Silva et al. 2019); in future, unsupervised machine and deep learning approaches may also help predict the molecular components of complex plant–pathogen interactions (reviewed by Yan and Wang 2022). However, collection and integration of high-quality, standardized phenotype data will still most likely be critical to future success in fully elucidating the *B. napus-L. maculans* pathosystem, and in identifying useable sources of resistance for breeding. A combination of these approaches, coupled with an understanding of the unique challenges faced in different cropping environments, may allow us to develop sustainable blackleg stem canker management strategies for rapeseed crop production.
